# Combined exposure to noise and ototoxic solvents: contributions to the revision of legal limits based on the literature

**DOI:** 10.47626/1679-4435-2026-1489

**Published:** 2026-06-15

**Authors:** Marcos Jorge Gama Nunes, Osvaldo Luiz Gonçalves Quelhas, Maria de Lurdes Costa Domingos

**Affiliations:** 1 Universidade Federal Fluminense (UFF), Departamento de Engenharia, Niterói, RJ, Brazil

**Keywords:** hearing loss, ototoxicity, occupational exposure, noise, occupational.

## Abstract

Work-related hearing loss resulting from combined exposure to noise and ototoxic solvents remains underestimated in occupational prevention practices. Substances such as toluene, xylene, and styrene, which are widely present in industrial environments, potentiate auditory damage even at levels below the tolerance limits considered safe. By conditioning the implementation of the Hearing Conservation Program solely on the action level for isolated noise exposure, Brazilian legislation proves insufficient in addressing the complexity of contemporary occupational risks. This opinion article proposes a critical reflection on the current regulatory limits and advocates for the revision of occupational hearing health prevention policies. Integrated strategies are discussed, including the simultaneous monitoring of exposure to noise and ototoxic chemical agents, the revision of occupational exposure limits, the continuous training of professionals in the field, and the adoption of hierarchical control measures. Protecting workers’ hearing health requires more comprehensive approaches capable of recognizing the interaction among different risk agents and promoting truly safe work environments.

## INTRODUCTION

Occupational hearing loss represents a major public health concern, affecting millions of workers regularly exposed to high noise levels across different industrial sectors [^1^]. Continuous exposure to sound levels above 85 dB(A) is particularly concerning, as it may cause irreversible damage to the hair cells of the inner ear. Furthermore, the duration of exposure over the years is a determining factor in the progression of hearing loss, establishing a direct relationship between noise intensity and the severity of the observed hearing impairment [^1^,^2^].

Recent studies in occupational toxicology have demonstrated that auditory damage is not limited to isolated noise exposure, highlighting the interaction between physical agents and ototoxic chemical substances [^3^]. Compounds such as toluene, xylene, and styrene, which are widely used in various industrial processes, significantly potentiate the harmful effects of noise on the auditory system, increasing the vulnerability of workers subjected to combined exposures [^4^,^5^]. Current evidence indicates that individuals simultaneously exposed to noise and ototoxic solvents are at greater risk of developing hearing loss when compared with those exposed only to noise [^6^,^7^].

In the Brazilian context, Regulatory Standard No. 7 reinforces the need for specific preventive measures within the scope of the Occupational Health Medical Control Program, with special attention to workers exposed to noise levels above 80 dB(A), particularly in situations involving concomitant exposure to ototoxic substances [^8^]. These measures include periodic audiometric testing, detailed environmental assessments, control of exposure to chemical agents, worker training, and continuous supervision regarding the proper use of personal protective equipment [^8^-^10^].

Despite the scientific recognition of the complexity of the factors involved in occupational hearing loss, preventive strategies remain predominantly focused on action levels related to isolated noise exposure, neglecting the interaction with other occupational factors, such as ototoxic solvents. Studies have demonstrated the occurrence of hearing loss even at concentrations below the currently regulated tolerance limits, highlighting the need for more integrated preventive programs [^1^,^5^,^11^,^12^]. Such programs should incorporate participatory and sector-specific approaches, including comprehensive occupational risk assessments and continuous monitoring of workers’ hearing health.

Therefore, it is essential that the Hearing Conservation Program (HCP) expand its scope by adopting more robust strategies for managing exposure to ototoxic substances, including medical evaluations at differentiated intervals and continuous reinforcement of guidance regarding the correct use of hearing protection equipment [^1^,^5^,^12^].

### NOISE

In current production processes, noise constitutes a physical agent widely present in workers’ daily activities, manifesting at different intensity levels and affecting work performance, quality of life, and health, in addition to increasing the risk of occupational accidents [^13^].

Occupational exposure to noise above tolerance limits may cause structural changes in the auditory system, leading to the development of noise-induced hearing loss. Its main characteristic is the progressive and irreversible degeneration of the hair cells of the organ of Corti, located in the inner ear, resulting in permanent hearing loss [^1^]. Recent evidence indicates that noise exposure, even at levels considered moderate, may cause hidden auditory synaptopathy, impairing speech comprehension even before elevation of conventional audiometric thresholds [^14^].

Noise-induced hearing loss is typically characterized as bilateral and generally symmetrical sensorineural hearing loss, initially affecting the higher frequencies (3, 4, or 6 kHz) and subsequently progressing to lower frequencies. It usually does not exceed 40 dB(HL) at low frequencies and rarely exceeds 75 dB(HL) at high frequencies [^8^]. Its progression tends to cease after interruption of exposure to intense noise.

The Brazilian Ministry of Labor and Employment warns that exposures above 100 dB(A) are extremely harmful and may cause significant auditory damage even with daily exposures of only 15 minutes [^10^]. Preventive measures therefore should be implemented when exposure exceeds 50% of the tolerance limit value, referred to as the action level.

For 8-hour work shifts, Brazilian legislation establishes 85 dB(A) as the maximum occupational noise exposure limit. National regulations adopt a 5 dB exchange rate, meaning that for every 5 dB increase in sound pressure level, the maximum permissible exposure time is reduced by half. In contrast, the National Institute for Occupational Safety and Health recommends a 3 dB exchange rate, considered more stringent and potentially more effective for protecting workers’ hearing health [^15^].

### OTOTOXIC SOLVENTS

Several volatile organic compounds used as industrial solvents have been associated with adverse effects on the auditory system. Due to their physicochemical properties, such as high volatility and low molecular weight, these agents may induce damage to the auditory pathways even at exposure levels below regulatory tolerance limits [^16^-^20^].

These compounds may be classified according to their toxicological relevance. Substances such as toluene, xylene, styrene, n-hexane, and trichloroethylene are considered high priority because of their ototoxic potential, whereas benzene, although present in several industrial formulations, is classified as lower priority [^21^,^22^]. Despite these differences, all share properties capable of compromising auditory sensory cells, as well as vestibular and central neural structures.

The presence of the “OTO” notation in the booklet published by the American Conference of Governmental Industrial Hygienists (ACGIH) represents an important warning regarding the potential of certain chemical substances to compromise workers’ auditory integrity, either through direct action on the auditory system or through synergistic interaction with noise exposure [^13^]. Occupational health and safety professionals are thus expected to be trained to implement control strategies integrating engineering and administrative measures. Furthermore, it is essential to adapt the HCP to the specific characteristics of the agents present in each occupational environment [^12^].

Chart 1 presents examples of solvents with recognized ototoxic potential [^13^], as well as their respective industrial applications, as described in the ACGIH booklet [^13^] as well as in the Guidelines and Minimum Parameters for the Development and Management of the Hearing Conservation Program published by Fundação Jorge Duprat Figueiredo de Segurança e Medicina do Trabalho [^8^].

**Chart 1:** List of chemical substances with ototoxic effects (OTO)

Substance	CAS registry number	Industrial applications
Toluene	108-88-3	Production of benzoic acid, benzaldehyde, explosives, dyes, and other organic compounds; solvent for paints, varnishes, gums, and resins; gasoline, naphtha; textiles and paper; artificial leather; detergent manufacturing.
Ethylbenzene	100-41-4	Styrene production; small portion used as a solvent.
n-Propylbenzene	103-65-1	Fabric dye; solvent for cellulose acetate.
Styrene	100-42-5	Manufacture of plastic, rubber, fiberglass; synthetic rubber; insulators; resins and plastics; agricultural products; stabilizers.
Methylstyrene	98-83-9	Production of polyester, resins, paints, and waxes.
Trichloroethylene	79-01-6	Solvent for organic materials; cleaning and degreasing agent.
p-Xylene	106-42-3	Manufacture of resins, paints, and varnishes; solvent for paints, coatings, adhesives, and rubber; aviation kerosene; surface protection; perfumes; repellents; epoxy; pharmaceuticals; leather.
n-Hexane	110-54-3	Cleaning of textiles, furniture, and leather; laboratory reagent; petroleum-derived products; solvent for vegetable oils.
Carbon disulfide	75-15-0	Diluent for iodine, sulfur, phosphorus, fats, vegetable oils, waxes, and rubber; widely used in industry and laboratories.

Source: Bittencourt, 2015 [^23^]; American Conference of Governmental Industrial Hygienists, 2024 [^13^].

CAS: Chemical Abstracts Service.

The concept of solvent-induced hearing loss refers to auditory impairment resulting from exposure to ototoxic solvents, regardless of the intensity of occupational noise [^3^,^16^]. Recent evidence demonstrates that auditory toxicity associated with these agents may occur even at exposure levels considered safe under current occupational action limits [^7^].

### COMBINED EFFECTS

In the context of occupational health, the concept of combined effect refers to the interaction between two or more risk agents acting simultaneously on the same physiological system, potentially resulting in amplified, modified, or aggravated effects compared with isolated exposure to each agent [^5^,^13^]. In the field of hearing, the association between noise and ototoxic solvents has become a growing concern, as these agents may compromise both peripheral and central auditory system structures [^7^,^16^].

The effects of exposure to noise and ototoxic solvents on hearing involve distinct and complementary mechanisms. Noise acts predominantly through mechanical injury and metabolic alterations in the hair cells of the cochlea, whereas solvents, absorbed through respiratory or dermal routes, are systemically distributed and affect neurosensory structures [^24^,^25^]. Substances such as toluene, styrene, and trichloroethylene have the potential to induce oxidative stress, apoptosis, and synaptic dysfunction which, in association with noise, result in potentiated effects, even at exposure levels below the established tolerance limits [^3^,^5^,^7^].

The ACGIH recognized these interactions by adopting the “OTO” notation in its occupational exposure limits booklet, identifying chemical substances with the potential to cause auditory impairment, either alone or in combination with noise [^13^]. This classification aims to guide the implementation of integrated preventive strategies, including the replacement of substances with less toxic alternatives, exposure control through engineering measures, limitation of exposure time, and inclusion of workers in HCP [^8^].

The study conducted by Ren et al. [^7^] demonstrated that the prevalence of hearing loss was higher among individuals simultaneously exposed to noise and solvents (53.6%) compared with those exposed only to noise (43.7%). These findings reinforce that hearing loss may occur even under conditions considered safe by current occupational exposure standards. The authors also observed that combined exposure intensifies the effects on the auditory system, increasing the risk of sensorineural hearing loss and producing more severe alterations than those resulting from isolated exposure to noise or solvents [^3^,^9^].

Understanding the combined effects between noise and ototoxic solvents is essential for redefining the criteria used to assess occupational auditory risk. The traditional approach, based exclusively on sound pressure levels, has proven insufficient in light of evidence showing that relatively low noise levels may produce more severe consequences when associated with chemical substances with ototoxic potential. In this context, Chart 2 demonstrates that knowledge of ototoxic concentrations capable of triggering occupational hearing loss mechanisms is indispensable for the integrated characterization of exposures and their biological effects.

**Chart 2:** Concentrations and exposure limits in the interaction between noise and ototoxic substances (OTO)

Agent	Concentration (ppm)	LEO ACGIH 2024 (ppm)	^*^Exposure result	Noise [dB(A)]	Study
Toluene	0.05	20	< 1% of the OEL	79.1	De Barba et al., 2005
Toluene	0.05	20	< 1% of the OEL	79.1	De Barba et al., 2005
Toluene	1.54	20	1%-10% of the OEL	83.0	Sliwinska-Kowalska et al., 2001
Toluene	2.23	20	10%-50% of the OEL	83.0	Sliwinska-Kowalska et al., 2001
Toluene	43.96	20	> 100% of the OEL	76.0-87.1	Metwally et al., 2012
Toluene	7.67	20	10%-50% of the OEL	74.1-90.3	Sliwinska-Kowalska et al., 2004
Toluene	31.45	20	> 100% of the OEL	86.7	Morata et al., 1993
Toluene	0.81	20	1%-10% of the OEL	87.7-93.0	Kim et al., 2005
Toluene	7.43	20	10%-50% of the OEL	73.2-88.6	Sliwinska-Kowalska et al., 2003
Toluene	0.90	20	1%-10% of the OEL	73.2-88.6	Sliwinska-Kowalska et al., 2003
Toluene	5.04	20	10%-50% of the OEL	81.2-84.3	Mohammadi et al., 2010
Toluene	25.00	20	> 100% of the OEL	86.3	Schaal et al., 2017
Xylene	0.05	20	< 1% of the OEL	79.1	De Barba et al., 2005
Xylene	0.05	20	< 1% of the OEL	79.1	De Barba et al., 2005
Xylene	6.52	20	10%-50% of the OEL	83.0	Sliwinska-Kowalska et al., 2001
Xylene	6.61	20	10%-50% of the OEL	83.0	Sliwinska-Kowalska et al., 2001
Xylene	59.11	20	> 100% of the OEL	76.0-87.1	Metwally et al., 2012
Xylene	56.47	20	> 100% of the OEL	74.1-90.3	Sliwinska-Kowalska et al., 2004
Xylene	19.64	20	50%-100% of the OEL	86.7	Morata et al., 1993
Xylene	0.57	20	1%-10% of the OEL	87.7-93.0	Kim et al., 2005
Xylene	31.55	20	> 100% of the OEL	81.2-84.3	Mohammadi et al., 2010
Xylene	89.36	20	> 100% of the OEL	81.2-84.3	Mohammadi et al., 2010
Xylene	3.00	20	10%-50% do LEO	86.3	Schaal et al., 2017
Ethylbenzene	1.89	20	1%-10% of the OEL	83.0	Sliwinska-Kowalska et al., 2001
Ethylbenzene	1.77	20	1%-10% of the OEL	83.0	Sliwinska-Kowalska et al., 2001
Styrene	22.40	10	> 100% of the OEL	60.0-72.5	Morioka et al., 2000
Styrene	1.10	10	10%-50% of the OEL	59.5-83.7	Morioka et al., 2014
Styrene	8.08	10	50%-100% of the OEL	73.2-88.6	Sliwinska-Kowalska et al., 2003
Styrene	14.06	10	> 100% of the OEL	73.2-88.6	Sliwinska-Kowalska et al., 2003

* Exposure result: relationship between the measured concentration of the chemical agent (expressed in ppm) and the respective OEL, presented as a percentage to facilitate interpretation of the degree of occupational exposure.

ACGIH: American Conference of Governmental Industrial Hygienists; dB: decibel; OEL: occupational exposure limit.

Although many concentrations of ototoxic solvents remain below 50% of the occupational exposure limit (OEL), established by the ACGIH at 10 ppm and, in Brazil, at 39 ppm, studies have demonstrated the occurrence of hearing loss in workers exposed to these agents [^7^]. Evidence indicates that ototoxic effects may be observed even at levels below 1% of the OEL, especially when associated with noise exposures considered safe, highlighting important limitations of current regulatory parameters for combined exposures [^26^].

Ren et al. [^7^] observed that 57.8% of individuals simultaneously exposed to noise and solvents presented hearing loss, with a 1.76-fold greater risk compared with the other groups, reinforcing the potential synergistic effect between chemical and physical agents.

Institutions such as Safe Work Australia recommend reducing the occupational noise exposure limit to 80 dB(A)/8h in the presence of ototoxic substances, whereas the ACGIH suggests 20% of the tolerance limit value as a reference for agents classified as ototoxic [^25^].

This reality highlights a critical limitation of the current regulatory approach: exposure levels considered acceptable do not always guarantee effective protection against auditory damage in environments with synergistic exposures. In this context, the professional responsible for HCP management must understand that the initiation of preventive actions cannot be conditioned exclusively on reaching 50% of the OEL. In practice, the mere presence of substances with ototoxic potential, even at relatively low concentrations, already requires specific preventive measures. Ignoring this interaction means perpetuating scenarios of occupational illness. Therefore, revising the parameters used to trigger preventive measures in HCP is not only a technical necessity but also an ethical and public health obligation.

## CONCLUSIONS

The present analysis demonstrates that work-related hearing loss resulting from combined exposure to noise and ototoxic solvents constitutes a relevant occupational risk that remains widely underestimated. Recent evidence demonstrates the occurrence of hearing loss even at exposure levels below those currently considered safe by Brazilian regulations, which establish the implementation of HCP upon reaching the action level (39 ppm). These findings reinforce the inadequacy of current regulatory criteria for the proper protection of workers’ hearing health.

The reformulation of HCP becomes necessary, incorporating the simultaneous monitoring of exposure to noise and ototoxic chemical agents. The persistence of fragmented preventive approaches is incompatible with the complexity of the risks present in contemporary occupational environments.

In this context, the adoption of integrated preventive measures is recommended, including the implementation of engineering controls, administrative measures, and appropriate personal protective equipment, as well as periodic audiometric examinations at reduced intervals, continuous training of professionals from the Specialized Service in Safety Engineering and Occupational Medicine, and the careful revision of the occupational tolerance limits currently adopted.

Preserving workers’ hearing health requires the implementation of comprehensive and continuous preventive measures based on the recognition of the interaction among the multiple risk factors present in contemporary work environments.

## Data Availability

The data supporting the findings of this study are available within the article
